# Biological pathways mediating the relationship between climate change, environmental pollutants, and severe mental disorders: a systematic review

**DOI:** 10.1192/j.eurpsy.2025.400

**Published:** 2025-08-26

**Authors:** P. Catapano, S. Cipolla, N. P. Sallusto, C. Lamberti, R. Murolo, G. Sampogna, M. Luciano

**Affiliations:** 1 Department of Psychiatry, University of Campania “Luigi Vanvitelli”, Naples, Italy

## Abstract

**Introduction:**

Climate change and pollution are deeply interconnected phenomena that pose significant risks to overall health, given their proven impact on the functionality of various human organs and systems. With increasing urbanization, global toxicity is expected to rise in the near future. It is therefore crucial to understand how environmental pollution affects mental health, particularly in rapidly growing urban areas.

**Objectives:**

This systematic literature review aims to explore the biological mechanisms linking exposure to environmental pollutants, climate change, and the onset and/or exacerbation of severe mental disorders.

**Methods:**

A search was conducted in the PubMed, Scopus, and APA PsycInfo databases, following PRISMA guidelines. Studies on humans and animal models examining the association between environmental pollutants, climate change, and mental disorders were included. A total of 48 articles were considered, comprising studies on humans (16 studies) and animal models (31 studies), along with one article that included both models.

**Results:**

Human studies revealed that exposure to particulate matter (PM 2.5 and PM10) increases the risk of depression and psychotic relapses through mechanisms involving inflammation, oxidative stress, and disruption of the hypothalamic-pituitary-adrenal axis. In animal models, pollution was shown to impact brain function by activating inflammatory responses, causing oxidative stress, damaging the hippocampus, and dysregulating neurotransmitters. Additionally, one study highlighted that climate change is associated with mood disorders by inducing changes in gene expression and psychophysical adaptation responses.

**Conclusions:**

The findings indicate that environmental pollutants and climate change can affect human mental health through complex biological pathways. Understanding these mechanisms is essential for developing prevention and intervention strategies. The One Health approach, which recognizes the interconnectedness of human, animal, and environmental health, is crucial for addressing the challenges posed by climate change and pollution.

**Disclosure of Interest:**

None Declared

